# Companions in the Abyss: A Feasibility and Acceptability Study of an Online Therapy Group for Healthcare Providers Working During the COVID-19 Pandemic

**DOI:** 10.3389/fpsyt.2021.801680

**Published:** 2022-01-14

**Authors:** Lorraine Smith-MacDonald, Jaimie Lusk, Dayna Lee-Baggley, Katherine Bright, Alexa Laidlaw, Melissa Voth, Shaylee Spencer, Emily Mack, Ashley Pike, Chelsea Jones, Suzette Bremault-Phillips

**Affiliations:** ^1^Department of Occupational Therapy, University of Alberta, Edmonton, AB, Canada; ^2^Department of Veterans Affairs, Portland Medical Center, Portland, OR, United States; ^3^Department of Family Medicine, Dalhousie University, Halifax, NS, Canada; ^4^Medical Centre, Department of Psychiatry, Leiden University, Leiden, Netherlands

**Keywords:** moral injury, healthcare provider (HCP), COVID-19, acceptance and commitment therapy, moral distress

## Abstract

**Introduction:**

In the context of the global pandemic of the SARS-CoV-2 coronavirus (COVID-19), healthcare providers (HCPs) have experienced difficult moral and ethical dilemmas. Research is highlighting the importance of moral injury (MI)–a trauma syndrome related to transgressing personal morals and values–in understanding the psychological harm and occupational impairment experienced by HCPs. To date, MI treatments have largely been developed for military personnel and veterans and rely on in-person one-on-one psychotherapy.

**Purpose:**

This project aims to explore the feasibility and acceptability of an evidence-informed online Acceptance and Commitment Therapy-based group therapy for MI in HCPs called “Accepting Moral Pain and Suffering for Healthcare Providers” (AMPS-HCP).

**Method:**

This feasibility and acceptability study included three separate phases with the first two phases focused on the development of the psychotherapeutic intervention and the third phase focused on the evaluation of the psychotherapeutic intervention. Eight participants (including registered nurses, practical nurses and respiratory therapists) completed seven 90-min sessions in an online group format. The focus of these sessions included ACT and MI psychoeducation and experientials. Qualitative semi-structured interview data was thematically analyzed while demographic and quantitative self-reported outcome data underwent descriptive analysis and non-parametric testing.

**Results:**

Results show that the intervention was highly feasible and acceptable to healthcare providers who worked on the frontline during COVID-19. Feasibility (referrals, eligibility, retention, participation engagement) was strong (8 out of 10 participants; 80% vs. desired >70% eligibility) and overall, 80% of participants completed 71% of the intervention. Data further supported the applicability and acceptability of the intervention. Preliminary data suggests that AMPS-HCP may supports HCPs to address MI.

**Discussion:**

This study is the first to report on the development and evaluation of an online MI group intervention for registered nurses, registered practical nurses, and respiratory therapists working during COVID-19. Results showed the use of both the online and group components of the intervention were acceptable and feasible during the third wave of COVID-19.

## Introduction

The global pandemic of the SARS-CoV-2 coronavirus (COVID-19) has placed untold strain and threat to global healthcare systems and healthcare providers (HCPs) (1). During the COVID-19 pandemic, HCPs across the globe have faced difficult moral and ethical decisions related to the enormous influx of patients with life-threatening conditions, resource limitations (e.g., ventilators, personal protective equipment, and life-saving medications), system overload (e.g., not having enough beds or HCPs to care for severely ill patients), policy changes, secondment, societal, and political stigma and denial, family needs (e.g., not allowing family to be present or say goodbye), exposure to mass death and dying, as well as personal elevated exposure risk for COVID-19 (2, 3). Additionally, HCPs have been stigmatized as vectors of contagion, resulting in their assault, abuse, and isolation during the COVID-19 pandemic (4, 5). This situation has caused many HCPs to feel helplessness, shame, anger, and guilt as hundreds of patients every day succumb to the illness (6, 7).

It is widely acknowledged that a large mental health crisis will be forthcoming for HCPs once the pandemic is over (8, 9). Significant research has been conducted regarding HCPs' experiences of other epidemics (e.g., SARS, MERS, Ebola) including within a Canadian context. For example, Maunder et al. (10) found that an estimated 29–35% of hospital workers experienced a high degree of stress, while another study found that 45% of nurses in Toronto during SARS experienced emotional distress (11). Other studies of HCPs during SARS have suggested rates of emotional distress being as high as 57% (12). Longitudinally, HCPs in Toronto reported significantly higher levels of burnout, psychological distress, and posttraumatic stress disorder (PTSD) 1–2 years post-SARS (13). More importantly, emotional and psychological distress experienced by HCPs was not necessarily associated exclusively with SARS trauma; rather it was compounded with issues related to quarantine, fear of contagion, concern for family, job stress, interpersonal isolation, perceived stigma, and conscription of non-specialists into infectious disease work (13).

### Moral Injury

Moral Injury (MI)–a specific trauma syndrome associated with the distress of witnessing or participating in acts that transgress personal morals, values, and beliefs (14)–has gained significant attention during COVID-19 particularly for HCPs (9, 15). One MI scholar commented that HCPs “are vulnerable to MI because (they) care” [(16), p. 1] and because they have faced innumerable moral and ethical dilemmas with no “right” solutions (15). While preliminary, research is highlighting the importance of MI when discussing the harm and impairment experienced by HCPs. O'Neal et al. (17) found 66.5% of surveyed HCPs felt moral distress related to conflicts between institutional constraints and what they believed was right during the pandemic. Similar moral dilemmas have been suggested for physicians who may be experiencing tensions between physicians' fidelity to best medical practices, their Hippocratic Oath, and managing scarce resources (18). Factors and experiences which have been found to cause moral distress during COVID-19 include uncertainty and lack of knowledge, fear of exposure, intra-professional tensions and miscommunications, policies that prevent or impede care, practicing within crisis standards of care, new roles/tasks and broken routines, and dealing with medical resource scarcity (19, 20). Noted emotions associated with COVID-19 related MI are feelings of overwhelm, fear, guilt, frustration, distrust, exhaustion, frustration, uncertainty, hopelessness, and helplessness (19).

While the long-term impacts of the coronavirus cannot be known at this time, MI is associated with significant mental health challenges, psychosocial issues, and occupational impairments. In a recent review, MI is associated with mental illnesses (e.g., PTSD, generalized anxiety disorder, major depressive disorder), physical health challenges (e.g., pain, sleep disturbances), behavioral issues (e.g., substance misuse, suicidal ideation) and occupational impairment (e.g., burnout, compassion fatigue, and work absenteeism) (21). Within COVID-19 literature, Wang et al. (22) explored prevalence and correlates of MI among physicians and nurses in mainland China during the pandemic and found an estimated 41.3% of HCPs screened positive for MI, with HCPs providing direct COVID-19 care to patients at 28% greater risk of MI. MI scores in these HCPs were also strongly and positively correlated with depression, anxiety, low well-being, and burnout symptoms (22). Similar results were found in a Canadian study where, again, moral distress significantly and positively predicted symptoms of depression, anxiety, PTSD, and burnout in HCPs (23). Universally, research is reporting that HCPs are at an increased risk for stress, burnout, and depression during the ongoing COVID-19 pandemic (24).

Central to the problem of MI is the lack of evidence-based treatment. To date, MI treatments have largely been developed for military personnel and veterans and rely on in-person one-on-one psychotherapy. Evidence-based, trauma-focused treatment approaches, such as Eye Movement Desensitization and Reprocessing, Prolonged Exposure, and Cognitive Processing Therapy, fail to directly address MI. Moreover, current scientific knowledge of MI highlights that this injury, while trauma-based, requires a different therapeutic approach (25, 26). For example, it has been suggested that rather than relying on strategies that address fear stimuli, MI is best resolved through expression of moral pain, mindfulness, self-compassion, grief and loss rituals, reparation of belief schemas, forgiveness, relationship repair, values or amends work, and self-care (27–31). A group approach to MI has also been suggested given the potential for group therapy to support relationship repair (32, 33). Meta-analyses into group therapy treatments have shown large and significant pre-post treatment reduction in PTSD symptoms (34, 35) and have also been shown to normalize symptoms, counteract isolation, provide peer support and observational learning, and ameliorate important shame-based cognitions (36)–all of which may be central to the treatment of MI.

### Acceptance and Commitment Therapy

Acceptance and Commitment Therapy (ACT) is a functional contextual cognitive behavioral psychotherapy approach that emphasizes mindfulness, acceptance, perspective-taking and values-based behavior change (37). ACT views human language as the core of many psychological disorders and human suffering (38) and seeks to bring language and thought under effective contextual control (39). Rather than trying to change the content of problematic thinking or the form and frequency of difficult private events such as unwanted thoughts, feelings, urges and sensations, ACT attempts to alter their psychological functions and influence on overt behavior by altering the socio-verbal context in which private events occur (39). The essential goal of ACT interventions is to increase capacity for behavior change by treating emotional avoidance, excessive literal response to cognitive content, and barriers to making and keeping commitments to value-based actions (38). This ability to choose to do what works in order to move toward who or what is important to the individual is known as *psychological flexibility* (40). Six processes combine to promote psychological flexibility and regulate patterns of behavior: cognitive defusion, acceptance, committed action, values, contact with the present moment and self-as-context (40).

Importantly, ACT has been theoretically suggested as a potentially ideal therapeutic modality for MI because of its therapeutic focus on cognitive flexibility, mindfulness, and value-driven behavior (28, 41). Specific to MI, ACT has been conceptualized as supporting the cultivation of acceptance of moral pain in the service of one's values instead of challenging the content of that pain (42). Theoretically, ACT posits that healing from MI requires a willingness to feel moral pain in the service of creating meaning, purpose, and vitality, while simultaneously reengaging areas of life (e.g., relationships, spirituality, and self-care) that are often ignored by those suffering a MI (43). Borges et al. (44) have argued that acceptance may be particularly important during the COVID-19 pandemic as challenging the content of painful experiences can pathologize the often functional response of moral pain. ACT for example has been explored through case studies for veterans with MI (45) and Evans et al. (42) wrote a self-guided workbook on MI using ACT; however, ACT has not been piloted in HCP populations, nor within an online group format for MI.

### Purpose

This project aims to explore the feasibility and acceptability of an evidence-informed online ACT-based group therapy for MI in HCPs, called “*Accepting Moral Suffering and Pain for Healthcare Providers”* (AMPS-HCP). As ACT has not been established as an evidence-based modality for MI, nor has a group approach or online format been trialed for a MI intervention, we determined to conduct a preliminary feasibility and acceptability study prior to embarking on a full scale randomized controlled pilot study.

## Methods

### Project Design

This pilot study included three separate phases with the first two phases focused on the development of the psychotherapeutic intervention and the third phase focused on the evaluation of the psychotherapeutic intervention. Mixed data collection was selected for the third phase as both quantitative and qualitative data are necessary to assess feasibility and acceptability.

#### Phase 1: Intervention Development

A systematic and critical review was conducted of the MI academic literature focused on MI interventions. Additionally, we consulted with 12 international MI Subject Matter Experts. The aim of this phase was to identify MI treatment approaches and components, along with potential benefits, barriers, and recommendations to the delivery of MI treatment via digital health platforms.

#### Phase 2: Intervention Construction and Training

Upon determining that no current intervention would be appropriate, the research team selected ACT as the evidence-based modality given its focus on value-driven behavior and grafted key MI constructs onto the six processes of ACT. This resulted in the development of a 100-page standardized clinician manual for AMPS-HCP. Training of registered mental health clinicians (i.e., psychologists, occupational therapists, psychotherapists) (*n* =4) occurred prior to delivery of the MI intervention. Two of the clinicians were recruited from the investigators listed on the grant.

#### Phase 3: Research of the Intervention

The AMPS-HCP intervention was delivered and researched for its feasibility and accessibility.

### Recruitment, Participants, and Setting

Potential participants were recruited via convenience and snowball sampling. An initial contact email with an electronic poster was sent to leaders within participating healthcare organizations asking them to forward the recruitment material. Additionally, given the need to recruit remotely, recruitment posts were placed on appropriate social media sites (with expressed consent). Potential participants were asked to directly contact the researchers and were subsequently screened for eligibility. If deemed eligible, participants were sent an electronic consent form to sign digitally via RedCap (an online data capturing platform) indicating their consent to participate in the intervention. Research Ethics Board and operational approvals were sought prior to commencing with the study. Participants were included if they were 18 years or older, spoke and understood English, were a registered nurse, registered practical nurse, or respiratory therapist (RT) who had been working during the COVID-19 pandemic, and felt they had been exposed to a potentially morally injurious experience. Ten potential participants initially expressed interest, meet inclusion criteria and we included in the study, but two dropped out before the intervention started due to personal reasons. No other participants were recruited as the recruitment material had been taken down (given fully the sample size in <48 hours) and the research team had informed the participating organizations that the study was closed. The final sample size was eight (*n* =8). The intervention was offered online via a Health Insurance Portability and Accountability Act compliant Canadian geo-fenced Zoom platform.

### Measures

#### Demographics

A demographics form was administered at baseline to assess age, gender, ethnicity, education, marital status, health profession, number of years in the profession, job title, and employment status.

#### Acceptability Measures

To assess for acceptability the Client Satisfaction Questionnaire (CSQ-8) (46) and Narrative Evaluation of Intervention Interview (NEII) (47) were used. The CSQ-8 is an eight-item, four-point Likert scale self-report measure that assesses overall satisfaction with the intervention being offered. The NEII is a 16-item semi-structured guide focused on the participants' perspectives of the impact of the intervention, helpful and unhelpful components, and comparison to other interventions. The semi-structured NEII interviews were conducted 1 week post completion of AMPS-HCP.

#### Feasibility Measures

Feasibility of AMPS-HCP was operationally defined as: sufficient patient referrals (ability to meet the minimum sample size of 8), eligibility (> 70% of potential participants meet the eligibility criteria), and enrollment (>50% of potential participants meet the eligibility criteria). The justification for the small sample size (*n* = 8) was two-fold: (1a) this was pilot feasibility and acceptability study to study what logistical challenges may be associated with offering the intervention, if potential participants felt the intervention adequately to address their needs, and what changes or modifications would be required in order for the intervention to be cultural/population appropriate; and (2) to ensure the sample size is representative of what would be used in a standard group therapy modality (approximately 6 to 10 participants). Evidence of feasible treatment delivery was defined as a minimum of 70% of participants completing 70% of the intervention. Regarding completion and retention, specific attention was given to the impact of shift-work and technology to the delivery of AMPS-HCP.

#### Fidelity Measure

Fidelity was established in terms of >80% adherence to clinician manual and ACT principles. Additionally, all four clinicians met for 30-min upon completion of each session to debrief, reflect, iteratively discuss changes to the previous and upcoming session, and to review the fidelity checklist.

#### Participant Treatment Outcomes Measure

Several measures of psychological health (PCL-5 and DASS-21), MI (MIOS), social function (MSPSS), occupational impairment (ProQoL), emotional regulation (DERS-18), coping (B-COPE), cognitive flexibility (AAQ), post-traumatic growth (PTGT), and resilience (CDRS-10) were administered pre-post intervention to help guide a future randomized controlled trial designed to assess the effect of AMPS-HCP (48).

### AMPS-HCP Intervention

The purpose of this transdiagnostic MI intervention was to support participants in cultivating acceptance of moral pain in the service of one's values rather than challenging the content of moral pain. The AMPS-HCP intervention consists of seven (one introductory and six therapeutic) 90-min online sessions administered over the course of consecutive weeks. Each session had the following structure: an opening poem/meditation, a review of the week using the Matrix (a tool help discriminate between internal and external experiences and identify actions that aligned with personal values), psychoeducation of an ACT principle, an integrative exercise, followed by psychoeducation of a MI principle, another integrative exercise to solidify learning and skill competence, and a closing poem/meditation. Time was allotted for individual and group reflections within each session to support learning and group cohesion. The therapeutic components of this intervention consisted of teaching six core processes within the sessions: (1) acceptance and self-compassion related to moral pain; (2) defusion related to self-criticism and resentment; (3) contact with the present moment, including contacting grief; (4) self-as-context and the role of meaning-making, narratives, and story-telling in perpetrating moral suffering; (5) contacting values related to moral injury, especially values behind our laments; (6) committing to value-driven actions of self-compassion and other reparative practices aimed to heal relationships with self-and/or others (see Table 1).

**Table 1:** Summary of AMPS-HCP sessions.

**Session**	**Intentions**	**Practical Content**
Introductory	Identify MI, potentially morally injurious experiences (PMIEs), and symptoms of MI. Explore how MI is related to violated values. Introduction of ACT and the Matrix as the framework for the sessions	- Introduction to ACT - Introduction to MI - Exercise reviewing PMIEs which may have occurred in COVID-19 - Introduction and review of the Matrix
One	Help participants identify the need for acceptance as the primary step toward healing of MI. Identification of the ways in which participants have been harmed during COVID-19, and the PMIE(s) which are most difficult to accept. Explore the role of compassion in helping to manage and accept moral pain	- Introduction to the concept of acceptance - Review of the Matrix - Exercise focused on identifying participants' MI monster (i.e., event that is most difficult to accept) - Education on compassion - Self-compassion meditation
Two	Help participants learn skills related to defusion and getting unstuck from negative or unwanted thoughts, emotions, and sensations. Emphasize the importance of viewing these as only thoughts, emotions, or sensations that will pass. Explore how PMIEs may impact and direct thoughts that further perpetuate suffering	- Introduction to the concept of fusion and defusion - Review of the Matrix - Exercise illustrating the attempts to remove “bad” thoughts - Education on how fused thoughts about PMIE(s) may be particularly difficult or sticky - The role of moral judgments in automatic or controlling thoughts - Exercise focused on defusion from the “inner dictator”
Three	Help participants to explore how they can stay in the present moment, and be more present and open to their thoughts, emotions, and sensations. Explore the intersectionality between grief and MI to show that MI includes loss because of the moral violation that occurred. Encourage participants to be open to grief and mourning the losses they have experienced while working during COVID-19	- Introduction of the concept of present moment awareness - Review of the Matrix - Present moment awareness exercise - Psychoeducation about loss and grief - Exercise focused on inviting in grief
Four	Help participants to see themselves as being within the current context of COVID-19, while also encouraging recognition for the larger more transcendent self. Exploration of the ways in which COVID-19 may have permanently or temporarily caused harm to the “self.” Utilize narrative and storytelling as a way to have participants begin to explore their individual MIEs and also frame those within the larger story of the pandemic	- Introduction to the concept of self-as-context - Review of the Matrix - Broken mirror exercise focused on illustrating the transcendent self - “I am” exercise - Exploration of the role of narratives/storytelling in MI - Exercise focused on writing COVID-19 MI story
Five	Help participants to continue exploring how MI or PMIE(s) may be impacting their behaviors and causing them to no longer be behaving in a value congruent manner. Help participants to continue exploring the ideas introduced by the “hero's journey” with specific attention given to the struggles of “ordeal in the abyss” and unresolved points of moral pain	- Introduction of the concept of values - Review of the Matrix - Exercise focused on encouraging value-driven behaviors - Psychoeducation on the role of meaning-making in MI - Exercise focused on writing COVID-19 lament
Six	Help participants to begin to explore how they could move to the “toward” side of the Matrix through value-driven behavior. Participants are encouraged to write down the values they have identified throughout the group as being harmed and to now match them to morally reparative behavior and action. Participants are reminded not to see these behaviors and actions as undoing their moral pain but allowing them to begin to re-experience vitality and meaning. Group wrap out and closing also occurs	- Review of the Matrix - Create a list of morally reparative behaviors - Explore thoughts, emotions, or sensations which might get in the way - Group wrap

### Data Collection

As the primary outcome of this study was to explore feasibility and acceptability of AMPS-HCP, semi-structured 45-min interviews via Zoom occurred ~1 week after completion of AMPS-HCP with participants. Interviews used the NEII questions to assess for acceptability of the study. They were then audio-recorded with permission and transcribed. Additionally, semi-structured 45-min interviews with the four clinicians providing AMPS-HCP were also conducted to assess for fidelity to the intervention and differences in opinions regarding feasibility and acceptability. The clinician weekly debrief notes and fidelity check-lists were also included as part of the data collection. To explore potential quantitative outcomes, REDCap was used to gather informed consent and the pre-post-questionnaire data.

### Data Analysis

Quantitative analysis included descriptive statistics (e.g., mean values, frequencies, and proportions) to summarize demographic data. Non-parametric analysis using Wilcoxon rank-sum was also conducted comparing pre-post differences within the participants. Qualitative data were thematically analyzed. Braun and Clarke (49) described thematic analysis as a method for identifying, analyzing, and reporting patterns (themes) in rich detail which may also allow for the researchers to interpret various aspects of the topic. Practically, thematic analysis involves examining the text in detail to identify recurring patterns (open coding) which are refined into “themes.” Initial codes for this study were developed through both deductive (i.e., based on acceptability and feasibility) and inductive coding (i.e., themes that emerged from the data itself). Four researchers reviewed the transcripts and independently coded the interviews to ensure the validity, reliability, and conformability of the analysis (50). The primary codes were then combined and tabulated into preliminary themes and reviewed by the research team. Upon completion of a second round of analysis, the proposed thematic theory underwent collective analysis by the entire research team, and key quotations were isolated to illustrate selected themes.

## Results

### Participant Demographics

The descriptive statistics of the participants are contained in Table 2. The average participant was middle-aged, Caucasian, female and had worked as a healthcare professional between 1 and 15 years.

**Table 2:** Participant demographics.

**Variable**	**Sub-variable**	**Percentage (*n*, %)**
Age (Average)		~37 years
Gender	Female	8 (100%)
	Male	0 (0%)
Ethnicity	Caucasian	7 (88%)
	African-Canadian	0 (0%)
	Latino	1 (12%)
	Asian	0 (0%)
	South Asian	0 (0%)
	First nation or metis	0 (0%)
Healthcare profession	Registered nurse	4 (50%)
	Licensed practical nurse	1 (12%)
	Respiratory therapist	3 (38%)
Highest level of education	High school	0 (0%)
	Diploma/college	3 (38%)
	Undergraduate degree	4 (50%)
	Graduate degree	1 (12%)
Years in the profession	First year in profession	0 (0%)
	1–5 years	4 (50%)
	5–10 years	0 (0%)
	10–15 years	3 (38%)
	15–20 years	1 (12%)
Employment status	Fulltime	5 (63%)
	Parttime	2 (25%)
	Causal	1 (12%)

### Feasibility (Referrals, Eligibility, Retention, Participation Engagement)

Within 1 week of recruiting the study was full (*n* = 10). All participants who volunteered to participate in the study were eligible to participate (10 out of 10 participants; 100% vs. desired >70% eligibility). None of the potential participants chose to decline to participate, and most participants asked if the study was open so that they could recruit friends and colleagues which we could not accommodate (10 out of 10 participants; 100% vs. desired >50% enrollment rate). Two participants withdrew from the study citing personal reasons for not being able to attend the sessions, but still asked to receive the weekly handouts as they found them to be helpful and beneficial to their mental health. The remaining eight participants completed the AMPS-HCP protocol. Four participants were able to complete all seven sessions (40% completed 100%), while three participants completed six of the seven sessions (30% completed 86%) and one participant completed four of the seven sessions (10% completed 57%). Overall, 80% of participants completed 71% of the intervention. The most common reason cited for not being able to attend was shift work or unexpectedly being called into work. It should also be noted that the intervention was offered to participants during the third wave of COVID-19 within Canada; some participants attended the intervention while on shift at the hospital during their breaks. Technology was not cited as being a barrier to attendance and as will be noted below, was found to be significant facilitator for attendance.

### Acceptability

All eight participants completed the NEII and seven of the eight participants completed the CSQ-8. The mean score of the CSQ-8 was 30 (the highest possible score being 34) and all participants rated the intervention as either “excellent” or “very good.” Qualitative thematic analysis further supported the acceptability and acceptability of the intervention. Specific sub-themes and supportive quotes are listed in the boxes below.

#### Theme 1: Applicability

Participants noted that the AMPS-HCP intervention was highly applicable to their experience of COVID-19. Many participants noted they were often expected as HCPs to “shove it down” or “deal with it” when mental health concerns arose. In particular, participants noted feeling failed by management as there was an expectation that frontline nurses and RTs would be able to manage on their own. Through engaging in the AMPS-HCP intervention, participants found they were allowed the space and validation to be “human beings” again and begin receiving the mental help they might not have found otherwise.

#### Theme 2: Usability

Participants expressed an openness and acceptability to engaging in the online group format of the intervention. They found the option of attending from home convenient as it allowed more flexibility within their work and home schedules and meant there was no time needing to be allocated toward travel. Participants also noted the online format increased their sense of comfort as they had more control over their environment and could turn off their microphones or cameras for increased privacy. When discussing the group format, many of the participants commented on the validation they were able to receive through knowing “*I wasn't the only one”* (P7) and the common verbalization aided them in “*analyzing and thinking things through”* (P5). One participant noted concerns about confidentiality as they had worked with some of the other participants, but expressed that this concern was not strong enough to impede their participation in the intervention.

#### Theme 3: Feasibility

Overall, feasibility did not arise as a significant issue for participants as most were able to attend most or all of the offered group sessions and found the online delivery accessible and convenient. The primary barrier participants referenced in attending the group was scheduling time around shift work (i.e., irregularity and inflexibility) which meant that some participants had to miss a session or two. In addition, some participants found the time the intervention was offered (5 pm) interfered with aspects of their personal or family lives, requiring them to make alternate arrangements for childcare or meal routines. Participants also noted that receiving information about the intervention (i.e., handouts or updates) via email was challenging given the number of emails they received every day during the pandemic.

#### Theme 4: Helpful Components

Participants also expressed that there were specific components of the AMPS-HCP intervention that were most helpful including: (1) permission to begin expressing the emotions they felt as a result of the pandemic; (2) a safe space to engage and unpack; (3) encouragement to begin to explore painful and distressing memories and emotions which they had otherwise tried to suppress; (4) a focus on application of the learned skills vs. straight psychoeducational content; and (5) the diversity of the facilitators which facilitate different styles and insights around session topics.

#### Theme 5: Outcomes

Participants generally found that participation in the group was beneficial to their mental health and in providing insight into further areas they want to work on. The participants commented on how the group helped them to gain awareness of the difficulties they were experiencing and to also gain the tools and resources to cope with these difficulties. In particular, participants found that realigning themselves with their values was highly beneficial. Several participants commented how they are more likely to seek out therapy in the future after participating in this group, as there are still things that they believe would be helpful for them to work through.

### Fidelity

A review of the fidelity checklist and debrief notes showed the facilitators were able to largely follow the standardized manual sessions per week (6 out of 7 weeks; 85% fidelity). However, the facilitators noted greater ability to maintain fidelity to the ACT content and exercises than the MI content and exercises. The facilitators noted that engaging in MI exercises required iterative adaptations both within and between sessions to effectively reflect the group process and honor the lived experiences of participants in the group. For example, 1 week's content shifted from reconciliation/forgiveness work to honoring values, coping amid current struggles and unknowns, taking inventory of losses and betrayals, and finding ways to accept difficult feelings. Other minor changes to the standardized manual included moving some of the psychoeducational content into the handouts rather than in the session content to allow for more time for the exercises and group discussions, the addition of more images, and the streamlining of metaphors throughout the manual so that these could be built upon each week. A full analysis of the facilitators' perspective will be forthcoming.

### Evaluation of Participant Outcome Measures

This study was not designed to test or ascertain the efficacy of the interventions, and non-parametric testing was limited because of the extremely small sample size. Self-reported questionnaires used were statistically insignificant with the exception of the DASS-21 (stress) subscale (*p* <0.05) (Table 3). However, trends toward significant were found for the DASS-21(total), DASS-21 (depression), the DERS-18 (emotional regulation), and the AAQ (cognitive flexibility). Additionally, while statistically insignificant, participants did (as an aggregate) have a 10 point decrease on the MIOS, indicating a potential reduction of MI symptoms.

**Table 3:** Participants self-reported outcomes.

	**Pre-mean**	**Post-mean**	**Change**	**Pre-SD**	**Post-SD**	***p*-value**
MIOS	68.63	58.75	9.88	18.08	32.59	0.401
PCL5	31.38	25	6.38	11.15	20.58	0.263
DASS21- total	25.13	17.25	7.88	5.36	15.22	0.159
DASS21-stress	11.75	5.43	6.32	2.76	5.13	0.018**
DASS21-anxiety	6.38	6	0.38	4.72	4.93	0.395
DASS21-depression	7	5.43	1.57	2.83	5.13	0.235
PROQoL	93.37	91.25	−2.12	9.71	6.18	0.612
DERS-18	42.63	32.50	10.13	11.95	21.2	0.161
BCOPE	69.63	56.38	13.25	9.86	36.02	0.674
AAQ	32.38	30	2.38	5.63	3.51	0.236
MSPSS	58.75	45.75	−13	32.59	29.36	0.499
CDR10	26.14	20.13	−6.01	3.723	12.85	0.397
PGTI	40.75	37	−3.75	10.33	29.49	0.674

## Discussion

The AMPS-HCP intervention is one of the first of its kind to explore the feasibility and acceptability of addressing MI using either a group or online format. These results are noteworthy given the overall lack of MI treatments, and that those currently being proposed predominately require one-to-one psychotherapy. Moreover, this is the first MI intervention to be developed exclusively for HCPs (nurses and RTs). While our results are preliminary, they showed that the AMPS-HCP was highly tolerated and meaningful, and participants perceived personal benefit to their mental health. Participants found the use of both the group and online format to be acceptable to them, and in some cases, perceived it as being more beneficial than if they had done it through in person one-on-one therapy. The need for novel evidence-based treatments cannot be overstated (51, 52) given the World Health Organization (1) statement that the mental health of HCPs is critical to successfully overcoming the pandemic. As feelings of being inadequately supported, morally compromised and helplessness may contribute to impaired mental health (53) and burnout (54, 55) the call to address MI as a key component of the COVID-19 related mental health crisis is high (56). Given this, the potential significance of AMPS-HCP should not be overlooked.

**Failed by Management/System**

“*The management does not listen. I don't feel cared for by management. We are told that this is what we signed up for and that we are to just deal with it. They are paying us and we should do a job. Death is a part of nursing. Yeah, I understand that but this feels very different. 19 deaths in 3 weeks. In a palliative unit things don't go that way. All my coworkers felt pressure.” (P2)*

“*Otherwise you're just always on the shove it down, suck it up, be more resilient, keep going, you can't break down, you gotta go to work.” (P7)*

“*There's a lot of times where we have to do things where we have no choice of where it comes from way above our heads and it's like you have to do this but we know it's not right. I think moral injury needs to be out there more and talked about and needs to be part of mental health training and everything like that...this should be a continuing thing that's offered, especially in the healthcare profession.” (P7)*

**Not Robots**

“*Yeah, we give so much of ourselves as health care workers, whether respiratory therapists or nurses. We know that our job is to just show up and do, we can just not do our jobs. But, at the same time we also are not robots. We are human beings and we have feelings and experiences.” (P6)*

“*We are always the helpers and now we need help. A lot of the doctors I hope that they are getting help. I don't know what they have, but the experience broke a lot of people.” (P2)*

**Accessible and Convenient**

“*I feel like it made it more accessible. It was like Ok I just have to block off one hour of time. I did not have to plan to drive to wherever the meeting is. That was kinda nice. Ok I am at my house, I have an internet connection. I just have to turn my computer on. So probably it made it easier in a way. Probably, if I had the option between the two now I would probably go online again for that factor.” (P4)*

“*I liked that at the end of the calls you're online and you're already at your house...You can just be at home and you can cry by yourself if you need to...That was a convenience thing more than anything, but also almost a safe thing. It made it feel a little more comfortable and a little more safe...And a lot of times when they were reading the poems I would turn off my camera and I would sit kind of like this just listening to it, just trying to get myself into the headspace of actually being able to be in the poem and I think if it was in-person I would maybe feel too self-conscious to do that.” (P3)*

**Group: Needing To Do It Together**

“*Whatever came out of everyone's mouth was exactly what I was thinking. Working on the unit was difficult. The things they said were difficult. Management did not take it seriously, they told us what staff signed up for. Like, this is normal. Just get through it. It is nice to see others that feel the same. We are all different areas and not the same hospital. It was nice to be validated. We have had none of that for a year and a half.” (P2)*

“*I think the group really helped me because it helped validate my own emotions and feelings and know that I wasn't the only one feeling that way” (P7)*.

**Shift Work**

“*My schedule is absolutely bonkers. I never have the same day off in a week. It's not regular in that way. It's days, nights, I flip all over the place… The main barrier is scheduling. That's the big one.” (P3)*

“*Work schedules are always the hardest part.” (P1)*

**Timing**

“*I struggled with childcare a little bit because of my husband's work schedule. Once I told my mother-in-law what I was doing, she made an effort to come and watch the kids so that I could do it. The timing was a bit weird because it started at 5pm. That is dinner time. I felt that it was a bit of a challenge. I made it to all of the sessions so that was not insurmountable” (P5)*.

While COVID-19 has caused a dramatic increase in the use of digital technology to provide mental health treatment, questions remain about the efficacy particularly for serious mental health conditions or vulnerable populations. As there is no literature to date on the use of digital health for MI, ensuring that an online delivery would not be problematic to participants was central to our study. Our results indicate that participants did not find it to be problematic or an impediment to MI treatment, thus supporting a growing body of literature which shows that online means may be useful for a number of serious mental health conditions including psychosis (57), PTSD (58), major depressive disorder (59), and anxiety disorders (60) along with vulnerable populations such as indigenous (61), refugee (62), and trauma-affected populations (63). Our results are similar to Samoocha et al. (64) that online digital health interventions could be empowering and facilitate greater involvement in the therapy.

Group therapy has been shown to be equally effective compared to individual treatment (65). Group therapy has also been noted to bring unique components to the therapeutic process not found in one-on-one. Yalom and Leszcz (66) noted that groups provide healing, bring hope, decrease isolation, and connect people to something larger than their own pain and loneliness. Group therapy has also been noted to be especially effective in addressing shame-based cognitions and emotions (67). Our intervention specifically refrained from encouraging participants to discuss details of specific incidents but on with instances experientially and working on changing relationships to the moral injury stories. By not focusing on details of participants PMIEs, there was considerable comradery, solidarity, and shared humanity in the suffering despite disparate settings, roles, and professions.

**Permission to Feel**

“*I feel it gave me the tools to reason out events in my life, identify feelings, or even gave me permission to feel my feelings. Make sense of things, differentiate between what are my thoughts and who I am and what are my values...I feel like I never really had those tools before.” (P1)*

**Digging into the Mental Garbage**

“*I just found it super helpful in dealing with emotions. I am just not the type of person that sits and thinks about my feelings or sits and thinks about what things were. And I think that a lot of girls from the group, or some of the girls in the group, definitely are, so I think the way they think through things, and then to actually sit and think through my stuff, I don't know that I would do it on my own. To sit and make that time and space where I think..I'm going to sit and deal with my mental garbage. For me, to sit aside that time and think, this is what I need to deal with my mental health.” (P5)*

**Shared Collective Trauma**

“*I was kind of surprised at how well it worked actually. And I think the reason, in my opinion, that it worked so well is because we all have a shared collective trauma, all of us. We all have been going through something similar. And we didn't talk specifically about which hospitals we worked at or what units we worked on but a lot of people on the call I think worked very closely with COVID patients either like on COVID units, in COVID ICUs or in hospitals that were accepting mass quantities of COVID patients.” (P3)*

**Very Little Doodling**

“*I would say my favorite thing about this group therapy format in particular is that every week it was very clear that the facilitators had something they wanted to teach and there was very little doodling in that regard. It was like okay let's talk about our weeks, poem, now we're going to teach you stuff, and now we're going to practice those things, and get out a pen and paper because now we're going to do a little activity. It was like every week there was kind of almost a to do list of things that we had to get through and be taught on the actual call which I thought was super super helpful that it was done during the session rather than stuff we had to do as homework and then come back next week with the actual homework done. So I thought that that was really good.” (P3)*

**Diverse Facilitators**

“*I think that the facilitators did an awesome job of…um…making it a really safe non-judgmental atmosphere. I really appreciated it, um… so we have 4 facilitators. And they were each very different styles and sort of different backgrounds and they all had unique insights, and different, you know, ways of speaking to things that were really helpful. So I definitely appreciated that. And all those years of experience, right, when are you ever going to get a group when you have so many really experienced and really brilliant therapists all in one room, right?” (P5)*.

**Momentum to Continue Therapy**

“*Oh 100% I am going to be looking for a therapist...It has helped me feel more comfortable to reach out to seek therapy. I didn't think I was going to like it—talking to other people.” (P2)*

“*I could have kept going a little more. I think there's just so much for everybody to work through.” (P7)*

“*But they felt heavy. Like in the other ones we learned other tools, but, I think the last two sessions, um, I think, well they kinda did. The second last session we came back and wrote a story. And then in the last session we wrote a shorter version of it, but I can't remember what it was called. That was helpful but they were quite heavy and I felt it would have been nice to have another session to break some of that down.” (P1)*

**Getting Back to My Values**

“*...Identifying values was probably one of the biggest things for me that I'll probably always reflect on what my values are and coming back to that…” (P1)*

“*I feel like I allow myself to feel my...feelings more. Like you know, if there is something sad at work. Or experience grief. I always felt before that it was not my place to feel that grief. And now I allow myself to feel that. If it is a sad situation, I am ok to feel sad even if it's a person I don't know. (I) definitely feel that I can allow myself to feel my feelings, happy, sad or whichever. Take time to reflect on them a little bit and instead of just, you know, brushing them off, or I used to find that I would keep myself busy and ignore all my feelings.” (P1)*

“*I still dread going to work some days, but I'm not absolutely miserable and anxiety ridden and full of fear and angst. I feel more calm in myself” (P7)*.

While group therapy has theoretically been suggested for MI because of these specific therapeutic components, little research has been done to validate its use. Our results suggest that group therapy may be a highly effective modality to use for MI. The use of an online format did not impact the ability to offer a group intervention. During COVID-19, particular focus has been given to the potential impact of video conferencing on the therapeutic process; indeed, video conferences in times of COVID-19 seem to be accepted and perceived as helpful by patients and providers (68). Dehkordi et al. (69) developed online Balint groups for healthcare professionals working on the frontlines of COVID-19 and found statistically significant decreases in anxiety and increases resilience scores in participants. These results highlight the growing evidence to support the use of online group therapy including for MI both during and potentially after COVID-19 (70, 71).

This study also provides useful information regarding MI and HCPs. Initially the researchers wondered if participants would relate to the concept of MI given the previous focus on moral distress especially within nursing literature (72). Participants quickly identified with the concept of MI as cycles of problematic thinking (e.g., avoidance of thoughts); emotions (e.g., judgement of or escape from emotions like anger, worry, hurt, sadness) and behaviors (e.g., disconnecting from relationships, reducing self-care). While participants identified with MI, it is important to note that they identified less with perpetration-based MI (self or others) and more with betrayal-based MI (73). Betrayal-based PMIEs included institutional neglect/dismissal, healthcare leadership dismissal of psychological harm, conflicting health policies, resource disparities resulting in a suboptimal care, “COVID denial,” non-compliance with masks/social distancing, personal exposure to COVID-19 (with long term effects), bearing the brunt of familial or society anger, resent or hostility; and witnessing painful deaths without the ability to comfort. As such, guilt and shame did not figure as prominently in this group as sadness, resentment, and hurt. This is interesting given the predominate focus on guilt and shame as the primary emotions tied to MI (74). Understanding the role of moral emotions more broadly may support a more comprehensive understanding about MI (28).

Key learnings were also gleaned regarding topics for the treatment of MI in HCPs. Ambiguous loss and disenfranchised grief were important concepts. Studies are currently lacking regarding the potential interplay or overlap of grief and MI; though some MI researchers have highlighted the potential grief elements of MI (75). Interestingly, the concept of forgiveness was not addressed as it seemed like it would be premature for some, and not a central issue for others; however, the struggles related to acceptance and self-compassion of moral pain were meaningful (29, 42). This may be an important distinction in treatment between preparation-based and betrayal-based MI. Additionally, it was clear that participants needed tangible practices and skills to be able to integrate, process and move through their moral pain. For example, the Matrix (76) helped to organize discussion, capture painful experiences, clarify values, and support participants in “toward” vs. “away” behavioral moves. Additionally, the use of storytelling, lament, expressive writing and poetry was also noted by participants to be particularly meaningful. The use of such mediums has been noted elsewhere as being potentially fruitful in the healing of MI and PTSD (36, 77). These results point to the importance of moving away from a strictly cognitive-based approach for treating MI and instead use language, metaphor, story, imagery, and spiritual practices to move people into their moral wounds (78, 79). Conversations around MI involve some of the most difficult and unanswerable spiritual and existential questions and require a very different approach than what is seen in traditional trauma therapy.

Further works is therefore warranted for AMPS-HCP. This should include a mixed-methods multisite randomized waitlist-controlled pilot study focused on exploring the efficacy of AMPS-HCP. In particular, it may be helpful to randomize severely affected COVID-19 healthcare sites as this would allow for greater comparability and assist in recruiting a statistically powered sample size of RNs, licensed practical nurses and RTs to further investigate the merit of AMPS-HCP as an evidence-based intervention. Given the larger sample size specific attention would be given to GBA + considerations (e.g., gender, ethnicity, minority status). Additionally, if the results from a AMPS-HCP pilot study were positive, care will be taken to explore implementation science processes to support scale and spread of the intervention.

### Limitations

There were several limitations of the current study that should be taken into consideration when interpreting the findings. First, the sample size is extremely small, and therefore generalizability is low. Second, participants were also recruited via convenience and snowball sampling and were therefore self-selected. This self-selection could mean that participants who were part of the sample were those who most identify with being morally injured because of COVID-19 or who were open to receiving treatment and support. The sample was also largely homogeneous with participants representing registered nurses, registered practical nurses, and RTs respiratory therapists. Additionally, all participants identified as female challenging the research team's ability to explore inclusion, diversity and equity principles; this should be addressed in future randomized control trials. Fourth, the sample size was not powered nor large enough to determine intervention effect, nor were the found effects explored longitudinally to determine sustainability. Fifth, as some participants were not able to attend all intervention sessions and did not complete all of the standardized outcomes measures this may have influenced the quantitative results. Sixth, it has been widely acknowledged in the literature that standardized questionnaires for MI are poor, and may be lacking in reliability, validity, and sensitivity (80). The MIOS was selected as the best questionnaire at the time of study construction particularly because it is not military-centric, however, caution should be warranted regarding if this questionnaire fully captured the causes, symptoms and harm caused by MI in HCPs. As this area of research is rapidly developing, there may exist other outcome measures for MI that may be appropriate for this population and exhibit improved reliability, validity, sensitivity, and temporal stability. Seventh given the conceptual challenges associated with MI, it is possible that data collection may not have fully encapsulated the events and processes that subsequently produced the noted harms associated with COVID related-MI. Finally, it is possible that participants were grateful to the researchers for receiving support and treatment, and therefore did not wish to express negative opinions regarding the intervention which will need to be countered (e.g., having greater separation between the facilitators of AMPS-HCP and the research team).

## Conclusion

This study is the first to report on the development and evaluation of an online MI group intervention for registered nurses, registered practical nurses, and RTs working during COVID-19. Results from this study showed the use of both the online and group components of the intervention were acceptable and feasible during the third wave of COVID-19. Moreover, participants identified strongly with the concept of MI and expressed the benefit and need for ongoing support to process the morally injurious experiences they had been exposed to in their work. As COVID-19 continues, there is an urgency to provide evidence-informed MI interventions which are tailored to address the unique needs of healthcare providers (HCPs) and the realities of COVID-19. Building on this feasibility and acceptability study, future research to explore and test AMPS-HCP seems warranted. Without this, healthcare systems risk that their most precious resource–their highly trained staff–will succumb to occupational injuries, mental illnesses, MI, or burnout. Fundamentally, when essential HCPs are doing well and are able to maintain health, safety, and security, all Canadians stand to benefit.

## Data Availability Statement

The data are not publicly available due to their containing information that could compromise the privacy of research participants. Requests to access the datasets should be directed to smithmac@ualberta.ca.

## Ethics Statement

The studies involving human participants were reviewed and approved by University of Alberta, Research Ethics Board, Pro00106350. The participants provided their written informed consent to participate in this study.

## Author Contributions

LS-M, JL, DL-B, and SB-P all equally participated in the conceptualization, development, delivery of the AMPS-HCP intervention, and also engaged in the original and final manuscript write-up. KB, AL, MV, SS, EM, AP, and CJ were involved in the data collection and analysis components of the research project and contributed to the writing and editing of the final manuscript. All authors agreed upon the authors order and attest to their involvement in the research project.

## Funding

This work was supported by the Government of Canada Innovation for Defense Excellence and Security (IDEaS) Grant #CPCA-0626-GUAlberta.

## Conflict of Interest

The authors declare that the research was conducted in the absence of any commercial or financial relationships that could be construed as a potential conflict of interest.

## Publisher's Note

All claims expressed in this article are solely those of the authors and do not necessarily represent those of their affiliated organizations, or those of the publisher, the editors and the reviewers. Any product that may be evaluated in this article, or claim that may be made by its manufacturer, is not guaranteed or endorsed by the publisher.
